# Probabilistic threshold analysis by pairwise stochastic approximation for decision-making under uncertainty

**DOI:** 10.1038/s41598-021-99089-z

**Published:** 2021-10-04

**Authors:** Takashi Goda, Yuki Yamada

**Affiliations:** School of Engineering, University of Tokyo, 7-3-1 Hongo, Bunkyo-ku, Tokyo, 113-8656 Japan

**Keywords:** Health care economics, Applied mathematics, Statistics

## Abstract

The concept of probabilistic parameter threshold analysis has recently been introduced as a way of probabilistic sensitivity analysis for decision-making under uncertainty, in particular, for health economic evaluations which compare two or more alternative treatments with consideration of uncertainty on outcomes and costs. In this paper we formulate the probabilistic threshold analysis as a root-finding problem involving the conditional expectations, and propose a pairwise stochastic approximation algorithm to search for the threshold value below and above which the choice of conditionally optimal decision options changes. Numerical experiments for both a simple synthetic testcase and a chemotherapy Markov model illustrate the effectiveness of our proposed algorithm, without any need for accurate estimation or approximation of conditional expectations which the existing approaches rely upon. Moreover we introduce a new measure called decision switching probability for probabilistic sensitivity analysis in this paper.

## Introduction

### Background

Probabilistic sensitivity analysis is an attempt to provide a framework for evaluating how the uncertainty of input parameters propagates to the uncertainty of model outputs^1,2^. Let $\varvec{\theta }=(\theta _1,\ldots ,\theta _s)$ be a vector of input random variables and consider a model described by $Y=f(\varvec{\theta })$. Here the output *Y* can be also regarded as a random variable because of the uncertainty of the input $\varvec{\theta }$. A primary interest of probabilistic sensitivity analysis for this simple setting is to identify which input variable $\theta _j$ (or, which group of input variables) affects the variability of the output *Y* most or least significantly. Among various approaches for measuring the relative importance of each input variable, variance-based sensitivity analysis due to Sobol’^3,4^ has been found quite useful for this purpose. Assuming the independence between the input random variables $\theta _1,\ldots ,\theta _s$, the following analysis-of-variance (ANOVA) decomposition of a square-integrable function *f* holds:

$$\begin{aligned} f(\varvec{\theta }) = f_{\emptyset }+ \sum _{\emptyset \ne u\subseteq \{1,\ldots ,s\}}f_u(\varvec{\theta }_u), \end{aligned}$$

where we write $\varvec{\theta }_u=(\theta _j)_{j\in u}$ for a non-empty set $u\subseteq \{1,\ldots ,s\}$, and each term is recursively given by $f_{\emptyset }=\mathbb {E}_{\varvec{\theta }}[f(\varvec{\theta })]$ and

$$\begin{aligned} f_u(\varvec{\theta }_u) = \mathbb {E}_{\varvec{\theta }\setminus \varvec{\theta }_u}\left[ f(\varvec{\theta })\right] -\sum _{v\subset u}f_v(\varvec{\theta }_v),\quad \text {for}\quad \emptyset \ne u\subseteq \{1,\ldots ,s\}. \end{aligned}$$

Here we note that $f_{\emptyset }$ is a constant and each function $f_u$ depends only on a group of input variables $\varvec{\theta }_u$. Because of the orthogonality of these terms^5^, the variance of *f* can be decomposed as

$$\begin{aligned} \mathbb {V}_{\varvec{\theta }}\left[ f(\varvec{\theta })\right] = \sum _{\emptyset \ne u\subseteq \{1,\ldots ,s\}}\mathbb {V}_{\varvec{\theta }_u}\left[ f_u(\varvec{\theta }_u)\right] . \end{aligned}$$

This equality enables us to measure the relative importance played by a group of input variables $\varvec{\theta }_u$ in several ways. The famous examples are

$$\begin{aligned} \sum _{\emptyset \ne v\subseteq u}\mathbb {V}_{\varvec{\theta }_v}\left[ f_v(\varvec{\theta }_v)\right] \quad \text {and} \quad \sum _{\begin{array}{c} \emptyset \ne v\subseteq \{1,\ldots ,s\}\\ v\cap u \ne \emptyset \end{array}}\mathbb {V}_{\varvec{\theta }_v}\left[ f_v(\varvec{\theta }_v)\right] =\mathbb {V}_{\varvec{\theta }}\left[ f(\varvec{\theta })\right] -\sum _{\emptyset \ne v\subseteq \{1,\ldots ,s\}\setminus u}\mathbb {V}_{\varvec{\theta }_v}\left[ f_v(\varvec{\theta }_v)\right] , \end{aligned}$$

where the first one measures the variance explained by $\varvec{\theta }_u$, whereas the second one measures the total variance minus the variance explained by the complement variables $\varvec{\theta }\setminus \varvec{\theta }_u$. In fact, there is a huge volume of literature on how to estimate these sensitivity measures^6–10^ and also on applications to real problems in various subjects^11–14^.

Looking only at the variability (or, the variance) of the output from a single model is not enough, however, if we are faced with a decision-making problem^15,16^. Let *D* be a finite set of possible alternative options for decision, and consider that each option $d\in D$ is associated with its model described by an utility function $Y_d=f_d(\varvec{\theta })$. In the context of health economic evaluations, for instance, *D* denotes the set of alternative treatments for a certain disease, $f_d$ represents the cost-effectiveness (or, the monetary net benefit) of each treatment $d\in D$, and the input variables $\varvec{\theta }$ include various unknown parameters related to the cost-effectiveness, such as the probability of side effect and the cost of treatment. Note that we assume that the set of input variables $\varvec{\theta }$ is common across all of the options and that the output $Y_d$ can be again regarded as a random variable because of the uncertainty of $\varvec{\theta }$. The fundamental problem here is two-fold:

1. to identity which option $d\in D$ is optimal under uncertainty on $\varvec{\theta }$, and
2. to identity which input variable $\theta _j$ (or, which group of input variables) affects the *variability of the optimal option* $d\in D$ most or least significantly.

Regarding the first problem, in the absence of any knowledge about $\varvec{\theta }$, the optimal option should be the one which maximizes the expected utility, i.e.,

$$\begin{aligned} d_{\mathrm {opt}}(\emptyset ) = \arg \max _{d\in D}\mathbb {E}_{\varvec{\theta }}\left[ f_d(\varvec{\theta })\right] . \end{aligned}$$

Throughout this paper, we assume that $d_{\mathrm {opt}}(\emptyset )$ is unique, that is, exactly one option achieves the maximum expected utility. In order to address the second problem above, the so-called *expected value of partial perfect information* (EVPPI) considers an ideal situation where the uncertainty on an individual variable or a group of variables can be removed completely, and evaluates how such a partially perfect knowledge on $\varvec{\theta }$ can lead to an optimal option different from the prior one $d_{\mathrm {opt}}(\emptyset )$ and yield an increment of the expected utility^17–20^. To be more precise, let us consider a partition of the components in the vector $\varvec{\theta }=(\varvec{\theta }_1,\varvec{\theta }_2)$. If we know the exact value of every component in $\varvec{\theta }_1$, the optimal option should be the one which maximizes the *conditional* expected utility given $\varvec{\theta }_1$, i.e.,

$$\begin{aligned} d_{\mathrm {opt}}(\varvec{\theta }_1) = \arg \max _{d\in D}\mathbb {E}_{\varvec{\theta }_2\mid \varvec{\theta }_1}\left[ f_d(\varvec{\theta })\right] , \end{aligned}$$

which can change depending on $\varvec{\theta }_1$. We note that, when $d_{\mathrm {opt}}(\varvec{\theta }_1)$ is not unique, that is, when several different options yield the same maximum conditional expected utility, the choice is arbitrary. Taking the average of the maximum conditional expected utility with respect to $\varvec{\theta }_1$, the EVPPI for $\varvec{\theta }_1$ is defined as its increment from the prior expected utility, i.e.,

$$\begin{aligned} \mathrm {EVPPI}_{\varvec{\theta }_1} = \mathbb {E}_{\varvec{\theta }_1}\left[ \max _{d\in D}\mathbb {E}_{\varvec{\theta }_2\mid \varvec{\theta }_1}\left[ f_d(\varvec{\theta })\right] \right] -\max _{d\in D}\mathbb {E}_{\varvec{\theta }}\left[ f_d(\varvec{\theta })\right] . \end{aligned}$$

The EVPPI takes a non-negative value and is bounded above by the expected value of perfect information (EVPI):

$$\begin{aligned} \mathrm {EVPI}= \mathbb {E}_{\varvec{\theta }}\left[ \max _{d\in D} f_d(\varvec{\theta })\right] -\max _{d\in D}\mathbb {E}_{\varvec{\theta }}\left[ f_d(\varvec{\theta })\right] . \end{aligned}$$

This way, it is indicated that the uncertainty of the random variables $\varvec{\theta }_1$ with a large EVPPI (close to EVPI) significantly affects the choice of the optimal option, whereas it is not the case for those with a small EVPPI. In fact, the equality $\mathrm {EVPPI}_{\varvec{\theta }_1}=0$ is equivalent to that $d_{\mathrm {opt}}(\varvec{\theta }_1)= d_{\mathrm {opt}}(\emptyset )$ happens almost surely (up to uniqueness of the argument), that is, the perfect knowledge on $\varvec{\theta }_1$ does not change the choice of the optimal option. This is how probabilistic sensitivity analysis can be performed for a decision model, and a strong interest in such decision-theoretic probabilistic sensitivity analysis can be found not only in health economic evaluations^21–24^ but also in petroleum engineering^16,25,26^. Here we emphasize that EVPPI is not the only measure for evaluating the relative importance of each input variable, and we shall introduce a new sensitivity measure called *decision switching probability* in this paper.

### What is probabilistic threshold analysis?

Based on the indication from EVPPI, it is natural to evaluate the threshold of $\varvec{\theta }_1$ around which the choice of the optimal option, $d_{\mathrm {opt}}(\varvec{\theta }_1)$, possibly changes. This is the aim of the so-called probabilistic parameter threshold analysis, which has been introduced quite recently as a way of probabilistic sensitivity analysis for decision-making under uncertainty^27^. Following the closely-related literature^27,28^, let us focus on the case where all of the input variables in $\varvec{\theta }$ are continuous and $\varvec{\theta }_1$ consists only of a single input variable $\theta _j$ for some $1\le j\le s$. Then the probabilistic parameter threshold for $\theta _j$, denoted by $K_j$, is simply defined as follows.

#### **Definition 1**

(*Probabilistic parameter threshold*) With the notation above, the probabilistic parameter threshold $K_j$ for an individual variable $\theta _j$ is defined by the set

$$\begin{aligned} K_j := \left\{ \theta _j \mid d_{\mathrm {opt}}(\theta _j)\,\text { is not unique}\right\} . \end{aligned}$$

**Figure 1:**
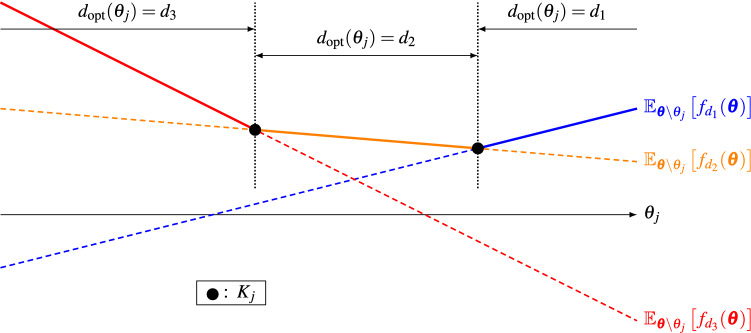
Schematic of the conditional expectations $\mathbb {E}_{\varvec{\theta }\setminus \theta _j}\left[ f_d(\varvec{\theta })\right]$ for different options $d\in D$ as functions of $\theta _j$, the conditional optimal option $d_{\mathrm {opt}}(\theta _j)$ and the probabilistic parameter threshold $K_j$.

Throughout this paper, we assume that the cardinality of $K_j$ is at most finite. Figure 1 shows a schematic of the probabilistic parameter threshold $K_j$ for the case $|D|=3$. The conditional expectation for each option $d\in D$ is drawn in a different color as a function of $\theta _j$. The optimal option $d_{\mathrm {opt}}(\theta _j)$ which maximizes the conditional expectation is equal to $d_3, d_2$ and $d_1$ in the left, middle and right intervals, respectively. The probabilistic parameter threshold $K_j$ consists of two intersection points in this example, with one between $d_3$ and $d_2$ and the other between $d_2$ and $d_1$.

#### *Remark 1*

It is obviously possible that $K_j$ is empty. In such a case, it implies from the continuity of $\theta _j$ that $d_{\mathrm {opt}}(\theta _j)$ does not change regardless of the value of $\theta _j$. Let us write $d'=d_{\mathrm {opt}}(\theta _j)$. If $d'\ne d_{\mathrm {opt}}(\emptyset )$ holds, the tower property of conditional expectations leads to

$$\begin{aligned} \mathbb {E}_{\varvec{\theta }}\left[ f_{d'}(\varvec{\theta })\right] = \mathbb {E}_{\theta _j}\mathbb {E}_{\varvec{\theta }\setminus \theta _j}\left[ f_{d'}(\varvec{\theta })\right] \ge \mathbb {E}_{\theta _j}\mathbb {E}_{\varvec{\theta }\setminus \theta _j}\left[ f_{d_{\mathrm {opt}}(\emptyset )}(\varvec{\theta })\right] = \mathbb {E}_{\varvec{\theta }}\left[ f_{d_{\mathrm {opt}}(\emptyset )}(\varvec{\theta })\right] , \end{aligned}$$

which contradicts our assumption that $d_{\mathrm {opt}}(\emptyset )$ is unique. Thus we must have $d'=d_{\mathrm {opt}}(\theta _j)\equiv d_{\mathrm {opt}}(\emptyset )$ for any $\theta _j$, leading to $\mathrm {EVPPI}_{\theta _j}=0$.

By definition, the probabilistic parameter threshold is not designed to measure the relative importance of each input variable for a decision model. Instead, it evaluates whether removing the uncertainty on $\theta _j$ completely can change the optimal option (for instance, from the prior one $d_{\mathrm {opt}}(\emptyset )$), and if so, that is, if $K_j$ is not empty, which values of $\theta _j$ make such change happen. This informs us of the following additional aspect for a decision problem, which cannot be captured only by the EVPPI. Suppose that both $\theta _i$ and $\theta _j$, with $1\le i<j\le s$, follow the standard normal distribution independently, and also that we have $\mathrm {EVPPI}_{\theta _i}\approx \mathrm {EVPPI}_{\theta _j}$. If $K_i=\{1\}, K_j=\{3\}, d_{\mathrm {opt}}(\theta _i)= d_{\mathrm {opt}}(\emptyset )$ for $\theta _i< 1$ and $d_{\mathrm {opt}}(\theta _j)= d_{\mathrm {opt}}(\emptyset )$ for $\theta _j< 3$, then, although an increment of the expected utility by knowing the exact value of either $\theta _i$ or $\theta _j$ is assumed almost the same each other, the chance of changing an optimal option $d\in D$ from $d_{\mathrm {opt}}(\emptyset )$ is quite different. For the variable $\theta _j$, such change happens when $\theta _j>3$, whose probability is only 0.0013, whereas it happens with probability 0.1587 for the variable $\theta _i$. Therefore, we can say that the variable $\theta _i$ is more sensitive to the variability of the optimal option than $\theta _j$.

Although our primary interest of this paper is in an efficient estimation of the probabilistic parameter threshold $K_j$, the above argument inspires us to introduce a related measure for decision-theoretic probabilistic sensitivity analysis as defined below. In what follows we call it decision switching probability.

#### **Definition 2**

(*Decision switching probability*) Let $\varvec{\theta }=(\varvec{\theta }_1,\varvec{\theta }_2)$ be a partition of the vector $\varvec{\theta }$. With the notation above, the decision switching probability for the variables $\varvec{\theta }_1$ is defined by

$$\begin{aligned} P_{\varvec{\theta }_1}:=\mathbb {P}_{\varvec{\theta }_1}\left[ d_{\mathrm {opt}}(\varvec{\theta }_1)\ne d_{\mathrm {opt}}(\emptyset ) \right] . \end{aligned}$$

In particular, for an individual parameter $\theta _j$, we simply write $P_j$ instead of $P_{\theta _j}$.

It is clear that the decision switching probability is defined as the probability of switching the optimal option $d_{\mathrm {opt}}(\varvec{\theta }_1)$ from $d_{\mathrm {opt}}(\emptyset )$ by knowing the exact values of $\varvec{\theta }_1$. This way, the decision switching probability can be useful in understanding which input variable a given decision-making problem under uncertainty is most (or least) sensitive to, measuring a decision-theoretic probabilistic sensitivity in a different way from the EVPPI. A connection between $K_j$ and $P_j$ is straightforward in that the domain of $\theta _j$ such that $d_{\mathrm {opt}}(\theta _j)\ne d_{\mathrm {opt}}(\emptyset )$ is determined by $K_j$, so that $P_j$ can be computed by using the (marginal) probability distribution of $\theta _j$. Moreover, as explained above, $\mathrm {EVPPI}_{\varvec{\theta }_1}=0$ is equivalent to $P_{\varvec{\theta }_1}=0$, as the latter means that $d_{\mathrm {opt}}(\varvec{\theta }_1)= d_{\mathrm {opt}}(\emptyset )$ happens almost surely. However, as discussed in Supplementary Information 1, the larger $\mathrm {EVPPI}_{\varvec{\theta }_1}$ does not necessarily mean the larger $P_{\varvec{\theta }_1}$ and vice versa, and hence, the decision switching probability can provide a complementary information to the existing decision-theoretic probabilistic sensitivity measure.

Regarding an estimation of the probabilistic parameter threshold $K_j$, a nested Monte Carlo approach is originally employed^27^. The computational procedure with the detailed input and output at each step is described in Algorithm 1. We can see that the algorithm takes a *double-loop* procedure with the outer loop for generating random samples of $\theta _j$ and the inner loop for generating random samples of $\varvec{\theta }_{-j}:=\varvec{\theta }\setminus \theta _j$ conditional to each sample of $\theta _j$, where *M* and *N* denote the numbers of inner and outer samples used, respectively. In the third item of Algorithm 1, the nominal choice of $\theta ^*_j$ is given by the midpoint $(\theta _j^{(n)}+\theta _j^{(n+1)})/2$. In order to reduce the necessary computational cost, a regression-based approach has been proposed^28^, which first approximates the inner conditional expectation $\mathbb {E}_{\varvec{\theta }_{-j}\mid \theta _j}\left[ f_d(\varvec{\theta })\right]$ by a regression model (as a function of $\theta _j$) and then applies a single-loop Monte Carlo sampling for $\theta _j$ to estimate $K_j$. However, these existing approaches rely upon accurate estimation (with large *M*) or approximation of inner conditional expectations, and both lack a theoretical support on convergence and computational complexity. 
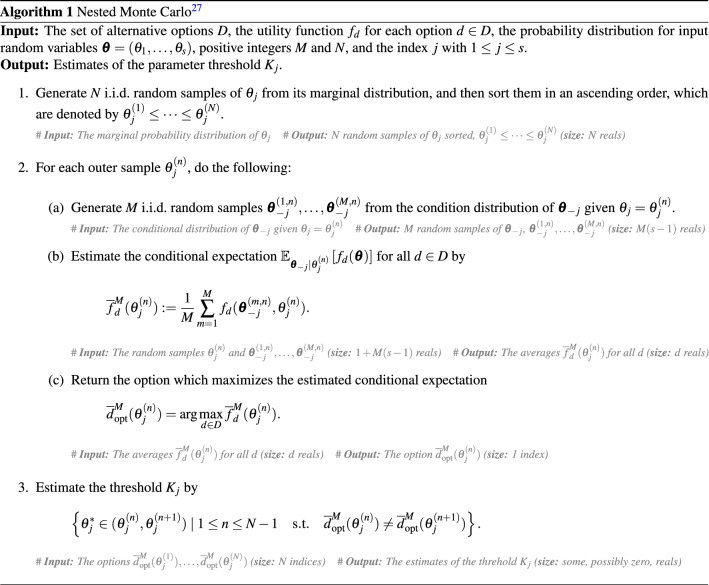


### Organization and contributions of this paper

Motivated mainly by applications to health economic evaluations, the aim of this paper is to develop an efficient algorithm to estimate the probabilistic parameter threshold $K_j$. We start from providing a formulation of the probabilistic threshold parameter analysis as a root-finding problem involving the conditional expectations. Then we propose a *pairwise stochastic approximation* approach to search for $K_j$ efficiently. The key difference from the existing approaches^27,28^ is that our proposed approach only requires an unbiased, but rough estimator of the inner conditional expectations and that the parameter threshold estimate is generated randomly only at the initial step and then updated iteratively. In fact, in our numerical experiments below, we use only one Monte Carlo sample to estimate the inner conditional expectations at each iteration step. Under some mild assumptions on $\varvec{\theta }$ and $f_d$’s, the standard theory on stochastic approximation from the literature^29–34^ directly applies to our proposed approach, so that the each element in $K_j$ can be found with a probabilistic error $\varepsilon$ typically by the computational cost of $O(|D|^2\varepsilon ^{-2})$. Numerical experiments for a simple synthetic testcase which compares three treatments and for a chemotherapy Markov model (see Supplementary Information 2 for the latter) both illustrate the effectiveness of the proposed approach. Finally we conclude this paper with some remarks. In summary, the main contributions of this paper are given as follows:

1. By reformulating the probabilistic threshold parameter analysis, an efficient pairwise stochastic approximation algorithm is proposed to estimate the probabilistic threshold.
2. Besides that our proposed algorithm is theoretically supported by the standard theory on stochastic approximation, numerical experiments for two examples confirm the effectiveness of our proposed algorithm and the superiority over the existing nested Monte Carlo method.
3. As discussed already, a new decision-theoretic sensitivity measure called decision switching probability is introduced in this paper, providing a complementary information to the existing measure EVPPI.

## Methods

### Formulation as a root-finding problem

Let us recall that, for a fixed value of $\theta _j$, the optimal option which maximizes the conditional expectation is denoted by

$$\begin{aligned} d_{\mathrm {opt}}(\theta _j) = \arg \max _{d\in D}\mathbb {E}_{\varvec{\theta }_{-j}|\theta _j}\left[ f_d(\varvec{\theta })\right] , \end{aligned}$$

and that our task is to find $\theta _j$ such that $d_{\mathrm {opt}}(\theta _j)$ is not uniquely defined. Let the set $\{d^1(\theta _j),d^2(\theta _j),\ldots ,d^{|D|}(\theta _j)\}$ be a reordering of the elements in *D* such that the inequality

$$\begin{aligned} \mathbb {E}_{\varvec{\theta }_{-j}|\theta _j}\left[ f_{d^1(\theta _j)}(\varvec{\theta })\right] \ge \mathbb {E}_{\varvec{\theta }_{-j}|\theta _j}\left[ f_{d^2(\theta _j)}(\varvec{\theta })\right] \ge \cdots \ge \mathbb {E}_{\varvec{\theta }_{-j}|\theta _j}\left[ f_{d^{|D|}(\theta _j)}(\varvec{\theta })\right] \end{aligned}$$

holds. Note that this ordering is arbitrary wherever the equality holds. With this notation, the fact that $d_{\mathrm {opt}}(\theta _j)$ is not unique is equivalent that the ordering of $d^1(\theta _j)$ and $d^2(\theta _j)$ is arbitrary, meaning that the corresponding conditional expectations are equal to each other. Hence, the probabilistic parameter threshold $K_j$ is equivalently given by

$$\begin{aligned} K_j&= \left\{ \theta _j \mid \mathbb {E}_{\varvec{\theta }_{-j}|\theta _j}\left[ f_{d^1(\theta _j)}(\varvec{\theta })\right] =\mathbb {E}_{\varvec{\theta }_{-j}|\theta _j}\left[ f_{d^2(\theta _j)}(\varvec{\theta })\right] \right\} \\&= \left\{ \theta _j \mid \mathbb {E}_{\varvec{\theta }_{-j}|\theta _j}\left[ \left( f_{d^1(\theta _j)}-f_{d^2(\theta _j)}\right) (\varvec{\theta })\right] =0 \right\} . \end{aligned}$$

Through this representation, our problem reduces to a root-finding problem which involves the conditional expectations. However, it is generally unknown which options correspond to $d^{1}(\theta _j)$ and $d^{2}(\theta _j)$, respectively, for given $\theta _j$.

Now, for two different options $d_1, d_2\in D$, we write

$$\begin{aligned} K_j^{(d_1,d_2)}= \left\{ \theta _j \mid \mathbb {E}_{\varvec{\theta }_{-j}|\theta _j}\left[ \left( f_{d_1}-f_{d_2}\right) (\varvec{\theta })\right] =0 \right\} . \end{aligned}$$

Note that $K_j^{(d_1,d_2)}$ can be the empty set if either

$$\begin{aligned} \mathbb {E}_{\varvec{\theta }_{-j}|\theta _j}\left[ \left( f_{d_1}-f_{d_2}\right) (\varvec{\theta })\right] >0 \quad \text {or}\quad \mathbb {E}_{\varvec{\theta }_{-j}|\theta _j}\left[ \left( f_{d_1}-f_{d_2}\right) (\varvec{\theta })\right] <0 \end{aligned}$$

holds for any $\theta _j$. It is obvious that we have $K_j^{(d_1,d_2)}=K_j^{(d_2,d_1)}$ and

$$\begin{aligned} K_j=K_j^{(d_1,d_2)}, \end{aligned}$$

if $$|D|=2$$, and

$$\begin{aligned} K_j \subseteq \bigcup _{\begin{array}{c} d_1,d_2\in D\\ d_1\ne d_2 \end{array}}K_j^{(d_1,d_2)}, \end{aligned}$$

if $|D|\ge 3$. Thus it suffices to search for the set $K_j^{(d_1,d_2)}$ for all the possible pairs $d_1,d_2\in D$ first and then to check whether each element in $$\bigcup _{\begin{array}{c} d_1,d_2\in D\\ d_1\ne d_2 \end{array}}K_j^{(d_1,d_2)}$$ is contained in $K_j$ or not. Note that the second step is not necessary for the case $|D|=2$.

Motivated by the formulation presented here, we below consider applying a stochastic approximation to find the roots of the conditional expectation $\mathbb {E}_{\varvec{\theta }_{-j}|\theta _j}\left[ \left( f_{d_1}-f_{d_2}\right) (\varvec{\theta })\right]$ and then propose a pairwise stochastic approximation approach to search for the threshold $K_j$, wherein some postprocessing based on a statistical hypothesis testing is required for the case $|D|\ge 3$ to see whether each element in $$\bigcup _{\begin{array}{c} d_1,d_2\in D\\ d_1\ne d_2 \end{array}}K_j^{(d_1,d_2)}$$ is contained in $K_j$ or not.

### Stochastic approximation for root-finding

Let $d_1,d_2\in D$ be two different options. In order to find the set $K_j^{(d_1,d_2)}$, i.e., the roots of the conditional expectation $\mathbb {E}_{\varvec{\theta }_{-j}|\theta _j}\left[ \left( f_{d_1}-f_{d_2}\right) (\varvec{\theta })\right]$, we use a stochastic approximation method. We refer to the book^35^ and the review article^36^ for a comprehensive information on stochastic approximation algorithms. In what follows, we briefly describe the stochastic approximation algorithm, as if the set $K_j^{(d_1,d_2)}$ contains only one element, which is denoted by $\tilde{\theta }_j^{(d_1,d_2)}$. Note that the resulting estimate will diverge if $K_j^{(d_1,d_2)}$ is empty, and also that several independent runs with different initial estimates are required if $K_j^{(d_1,d_2)}$ contains more than one element and all the elements are needed to be found.

For a fixed value of $\theta _j$ and $M\in \mathbb {Z}_{> 0}$, we denote by $\varvec{\theta }_{-j}^{(1)},\ldots ,\varvec{\theta }_{-j}^{(M)}$ the i.i.d. random samples of $\varvec{\theta }_{-j}$ conditional on $\theta _j$. Then the conditional expectation $\mathbb {E}_{\varvec{\theta }_{-j}|\theta _j}\left[ \left( f_{d_1}-f_{d_2}\right) (\varvec{\theta })\right]$ can be estimated unbiasedly by the following Monte Carlo estimator:

1 $$\begin{aligned} \overline{f_{d_1}-f_{d_2}}^M(\theta _j) := \frac{1}{M}\sum _{m=1}^{M}\left( f_{d_1}-f_{d_2}\right) (\varvec{\theta }_{-j}^{(m)},\theta _j). \end{aligned}$$

Then the classical Robbins-Monro algorithm^29^ searches for the solution $\tilde{\theta }_j^{(d_1,d_2)}$ by

2 $$\begin{aligned} \theta _{j}^{t+1} =\theta _{j}^{t}-\alpha _t\times \overline{f_{d_1}-f_{d_2}}^M(\theta _j^{t}) \end{aligned}$$

with an initial point $\theta _j^1$ and a sequence of decreasing step sizes $\alpha _1,\alpha _2,\ldots >0$. The initial estimate $\theta _j^1$ can be generated, for instance, randomly from the marginal probability distribution of the variable $\theta _j$. The well-known averaging technique, found independently by Polyak^32^ and Ruppert^33^, outputs the average

$$\begin{aligned} \Theta _j^t := \frac{1}{t}\sum _{u=1}^{t}\theta _{j}^{u}, \end{aligned}$$

instead of the nominal estimate $\theta _j^t$. In order to establish a convergence result of the estimate $\theta _j^t$ to $\tilde{\theta }_j^{(d_1,d_2)}$, Robbins and Monro originally consider the following assumptions^29^:

1. (conditional expectation)
  $$\begin{aligned} {\left\{ \begin{array}{ll} \mathbb {E}_{\varvec{\theta }_{-j}|\theta _j}\left[ \left( f_{d_1}-f_{d_2}\right) (\varvec{\theta })\right]<0 &{} \text {for}\, \theta _j<\tilde{\theta }_j^{(d_1,d_2)},\\ \mathbb {E}_{\varvec{\theta }_{-j}|\theta _j}\left[ \left( f_{d_1}-f_{d_2}\right) (\varvec{\theta })\right]>0 &{} \text {for}\, \theta _j>\tilde{\theta }_j^{(d_1,d_2)}.\\ \end{array}\right. } \end{aligned}$$
2. (conditional variance)
  $$\begin{aligned} \mathbb {V}_{\varvec{\theta }_{-j}|\theta _j}\left[ \left( f_{d_1}-f_{d_2}\right) (\varvec{\theta })\right] \le \sigma _j^2<\infty \end{aligned}$$
  holds for any $\theta _j$.
3. (step sizes)
  $$\begin{aligned} \sum _{t=1}^{\infty }\alpha _t=\infty \quad \text {and}\quad \sum _{t=1}^{\infty }\alpha _t^2<\infty . \end{aligned}$$

It is obvious that when the sign of the conditional expectation given in the first item is opposite, the recursion (2) should be replaced by

$$\begin{aligned} \theta _{j}^{t+1} =\theta _{j}^{t}+\alpha _t\times \overline{f_{d_1}-f_{d_2}}^M(\theta _j^{t}). \end{aligned}$$

It follows from the third item that the step sizes must decay at the order of $t^{-\alpha }$ with $1/2<\alpha \le 1$. As shown by Ruppert and Juditsky^34^, if the conditional expectation $\mathbb {E}_{\varvec{\theta }_{-j}|\theta _j}\left[ \left( f_{d_1}-f_{d_2}\right) (\varvec{\theta })\right]$ is linear in $\theta _j$, this condition can be relaxed to $0<\alpha <1$ by the Polyak-Ruppert averaging, which allows for more slowly decaying step sizes. Regarding the results on the convergence rates, we refer to Section 5 of the review article^36^ both for the standard Robbins-Monro iteration and for the Polyak-Ruppert averaging. Roughly speaking, the estimate ($\theta _{j}^{t}$ or $\Theta _{j}^{t}$) typically converges almost surely to $\tilde{\theta }_j^{(d_1,d_2)}$ with the rate of $1/\sqrt{t}$ under mind assumptions on $\varvec{\theta }$ and $f_d$’s.

#### *Remark 2*

The stochastic approximation algorithm described above does work to search for the pairwise set $K_j^{(d_1,d_2)}$ for any sample size $M\ge 1$ in (1). Hence, by formulating the probabilistic threshold analysis as a stochastic root-finding problem, we can avoid the difficulty inherent to the nested structure considered in the literature^27,28^. Moreover, in order to improve the stability of the algorithm, we can apply some of variance reduction techniques including Latin hypercube sampling^37^ or (randomized) quasi-Monte Carlo sampling^38^, as long as the resulting estimator is unbiased as with the standard one (1).

### Search for parameter threshold

Having estimated the set $K_j^{(d_1,d_2)}$ for all the possible pairs $d_1,d_2\in D$, it suffices to check whether each element in the estimated set $K_j^{(d_1,d_2)}$ is contained in $K_j$ or not. Note again that this step is not necessary if $|D|=2$. For $|D|\ge 3$, we carry out this step by the following statistical hypothesis testing.

Let $\hat{\theta }^{(d_1,d_2)}_j$ be an element in the estimated set $K_j^{(d_1,d_2)}$. Then the null and alternative hypotheses are given by

$$\begin{aligned} H_0:\, \hat{\theta }^{(d_1,d_2)}_j\in K_j\quad \text {and}\quad H_1:\, \hat{\theta }^{(d_1,d_2)}_j\not \in K_j, \end{aligned}$$

respectively. The condition for the null hypothesis $H_0$ is equivalent that

$$\begin{aligned} \mathbb {E}_{\varvec{\theta }_{-j}|\hat{\theta }^{(d_1,d_2)}_j}\left[ \left( f_{d}-f_{d_1}\right) (\varvec{\theta })\right] \le 0 \quad \text {and}\quad \mathbb {E}_{\varvec{\theta }_{-j}|\hat{\theta }^{(d_1,d_2)}_j}\left[ \left( f_{d}-f_{d_2}\right) (\varvec{\theta })\right] \le 0 \end{aligned}$$

hold for all $d\in D\setminus \{d_1,d_2\}$, while the condition for the alternative hypothesis $H_1$ is equivalent that there exists at least one option $d\in D\setminus \{d_1,d_2\}$ such that either

$$\begin{aligned} \mathbb {E}_{\varvec{\theta }_{-j}|\hat{\theta }^{(d_1,d_2)}_j}\left[ \left( f_{d}-f_{d_1}\right) (\varvec{\theta })\right]> 0 \quad \text {or}\quad \mathbb {E}_{\varvec{\theta }_{-j}|\hat{\theta }^{(d_1,d_2)}_j}\left[ \left( f_{d}-f_{d_2}\right) (\varvec{\theta })\right] > 0 \end{aligned}$$

holds. Assuming the normality of the Monte Carlo estimators

$$\begin{aligned} \overline{f_{d}-f_{d_1}}^N(\hat{\theta }^{(d_1,d_2)}_j)\quad \text {and}\quad \overline{f_{d}-f_{d_2}}^N(\hat{\theta }^{(d_1,d_2)}_j) \end{aligned}$$

for all $d\in D\setminus \{d_1,d_2\}$ with large sample size *N*, for instance, the conventional one-sided *t*-test applies independently to each individual inequality null, and the null hypothesis $H_0$ will be rejected or not with some significance level.

The necessary cost of the hypothesis testing is considered moderate or even negligible as compared to that of estimating the set $K_j^{(d_1,d_2)}$ for all the possible pairs $d_1,d_2\in D$. Since the convergence results on the stochastic approximation method implies that each element in $K_j^{(d_1,d_2)}$ can be estimated with a probabilistic error $\varepsilon$ by the cost of $O(\varepsilon ^{-2})$, the total cost of our proposed approach to estimate the probabilistic threshold $K_j$ itself is of order $O(|D|^2\varepsilon ^{-2})$ (up to the cardinality of each pairwise set $K_j^{(d_1,d_2)}$), where the factor $|D|^2$ comes from the number of possible pairs $d_1,d_2\in D$, which is $|D|(|D|-1)/2$. The overall computational procedure of our proposed approach is summarized in Algorithm 2.
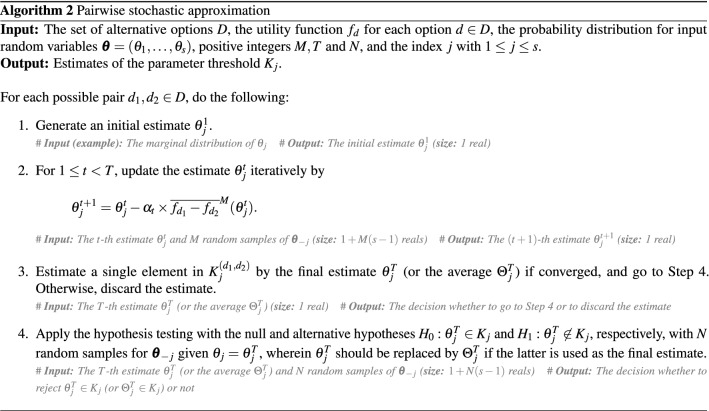


## Numerical experiments

To demonstrate the effectiveness of our proposed approach, here we conduct numerical experiments for a simple synthetic testcase^39^ comparing three medical treatments. In Supplementary Information 2, we present our numerical results for a more complicated chemotherapy Markov model introduced by Heath and Baio^40^.

### Model setting

The example we use here is taken from Hironaka et al.^39^, which extends the model originally introduced by Ades et al.^41^ in the context of medical decision making. As was explained^39^, the original version of this synthetic cost-effectiveness model compares only two treatments (some standard of care and a new treatment) on the prevention of a critical event, denoted by *E*, whereas three different treatments $D=\{d_1,d_2,d_3\}$ are compared in the extended model with $d_1$ being the standard of care and $d_2$ and $d_3$ being two different new treatments. The standard of care $d_1$, on the one hand, is cost-free and has no risk that the side effect (*SE*) occurs, while the probability that the critical event occurs is relatively large. The new treatments $d_2$ and $d_3$, on the other hand, are both costly and have some probabilities that the side effect occurs, while the probabilities of the critical event are relatively smaller than $d_1$.

Importantly, the above-mentioned costs and probabilities of the critical event and the side effect themselves are not known precisely, so that we model them as random variables. We refer to Table 1 for a detailed description on these model inputs. The utility function $f_d$ for each treatment $d\in D$ represents the monetary net benefit of *d* as a function of the input vector $\varvec{\theta }$ which consists of 12 individual random variables *L*, $Q_E$, $Q_{SE}$, $C_E$, $C_{SE}$, $C_{T,d_2}$, $C_{T,d_3}$, $P_{E,d_1}$, $OR_{E,d_2}$, $OR_{E,d_3}$, $P_{SE,d_2}$, $P_{SE,d_3}$, which are denoted by $\theta _1,\ldots ,\theta _{12}$, respectively, in this order. Furthermore, the model contains three constants $C_{T,d_1}, P_{SE,d_1}, \lambda$, while two parameters $P_{E,d_2}$ and $P_{E,d_3}$ are defined as functions of $P_{E,d_1}, OR_{E,d_2}$ and $P_{E,d_1}, OR_{E,d_3}$, respectively. Here, unlike the original model^41^, the extended model includes the correlations between odds ratios (OR) of the critical events ($OR_{E,d_2}$, $OR_{E,d_3}$), treatment costs ($C_{T,d_2}$, $C_{T,d_3}$), and probabilities of side effects ($P_{SE,d_2}$, $P_{SE,d_3}$), which makes the decision-making problem computationally harder.

Now the net benefit function $f_d$ for each treatment $d\in D$ is defined by

$$\begin{aligned} f_d(\varvec{\theta })&= P_{SE,d}P_{E,d}\left[ \lambda \left( L\frac{1+Q_E}{2}-Q_{SE}\right) -(C_{SE}+C_E)\right] + P_{SE,d}(1-P_{E,d})\left[ \lambda (L-Q_{SE})-C_{SE}\right] \\&\quad + (1-P_{SE,d})P_{E,d}\left[ \lambda L\frac{1+Q_E}{2}-C_E\right] + (1-P_{SE,d})(1-P_{E,d})\lambda L-C_{T,d}, \end{aligned}$$

where the first four terms correspond to possible four outcomes (whether or not the side effect occurs and whether or not the critical event occurs) and the fifth term denotes the cost of *d*. We note that the net benefit is expressed as a multi-linear function of most of the elements in $\varvec{\theta }$. However, $f_{d_2}$ and $f_{d_3}$ are both nonlinear with respect to $P_{E,d_1}, OR_{E,d_2}$ and $P_{E,d_1}, OR_{E,d_3}$, respectively, which makes it hard to compute the probabilistic parameter thresholds exactly for this model. As has been discussed, our interest is to infer which input parameter affects the choice of the optimal treatment more or least significantly.

**Table 1:** The input parameters involved in the synthetic testcase.

Description	Parameter	Distribution
Lifetime remaining	$L\, (\theta _1)$	*N* (30, 25)
QALY after critical event, per year	$Q_E\, (\theta _2)$	$\text {logit-normal}\left( 0.6, 1/36\right)$
QALY decrement due to side effects	$Q_{SE}\, (\theta _3)$	*N* (0.7, 0.01)
Cost of critical event	$C_E\, (\theta _4)$	$N(2\times 10^5, 10^8)$
Cost of side effect	$C_{SE}\, (\theta _5)$	$N(10^5, 10^8)$
Cost of treatment $d=d_1$	$C_{T,d_1}$	0 (constant)
Cost of treatments $$d=d_2,d_3$$	$$C_{T,d}$$$$(\theta _6, \theta _7)$$	$$N\left( \begin{pmatrix} 1.5\times 10^4 \\ 2\times 10^4\end{pmatrix}, \begin{pmatrix}300 &{} 100 \\ 100 &{} 500\end{pmatrix}\right) $$
Probability of critical event on treatment $d=d_1$	$P_{E,d_1}\, (\theta _8)$	$\text {Beta}(15, 85)$
Odds ratios of critical event relative to treatment $$d=d_1$$ $$\frac{P_{E,d}/(1-P_{E,d})}{P_{E,d_1}/(1-P_{E,d_1})}$$	$$OR_{E,d}$$$$(\theta _9,\theta _{10})$$	$$\text {log-normal}\left( \begin{pmatrix} -1.5 \\ -1.75\end{pmatrix}, \begin{pmatrix}0.11 &{} 0.02 \\ 0.02 &{} 0.06\end{pmatrix}\right) $$
Probability of critical event on treatments $d=d_2,d_3$	$P_{E,d}$	Derived from $P_{E,d_1}$ and $OR_{E,d}$
Probability of side effect on treatment $d=d_1$	$P_{SE,d_1}$	0 (constant)
Probability of side effect on treatments $$d=d_2,d_3$$	$$P_{SE,d}$$$$(\theta _{11},\theta _{12})$$	$$\text {logit-normal}\left( \begin{pmatrix} -1.4 \\ -1.1\end{pmatrix}, \begin{pmatrix}0.10 &{} 0.05 \\ 0.05 &{} 0.25\end{pmatrix}\right) $$
Monetary value of 1 QALY	$\lambda$	75,000 (constant)

Note that $\text {log-normal}(\mu ,\Sigma )$ and $\text {logit-normal}(\mu ,\Sigma )$ denote the log-normal and logit-normal distributions, respectively, with $\mu$ and $\Sigma$ being the mean vector and the covariance matrix of the corresponding normal distribution, respectively. $\mathrm {Beta}(\alpha ,\beta )$ denotes the Beta distribution with shape parameters $\alpha ,\beta >0$. The word QALY appearing in the first column stands for quality-adjusted life year.

## Results and discussion

### Reference results

**Figure 2:**
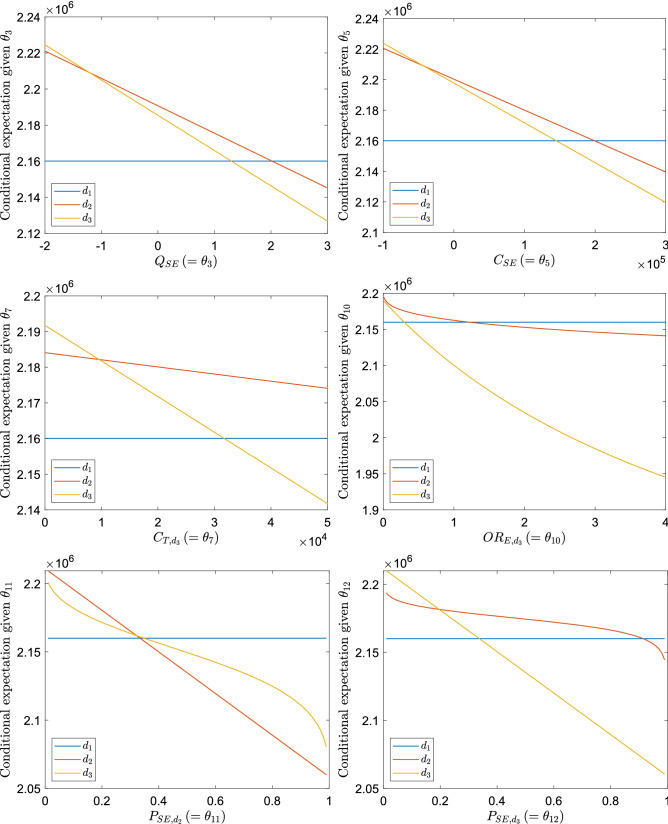
The conditional expectations $\mathbb {E}_{\varvec{\theta }_{-j}|\theta _j}[f_d(\varvec{\theta })]$ as functions of $\theta _j$ for $d\in \{d_1,d_2,d_3\}$. The results for $\theta _3$ (left top), $\theta _5$ (right top), $\theta _7$ (left middle), $\theta _{10}$ (right middle), $\theta _{11}$ (left bottom) and $\theta _{12}$ (right bottom) are shown respectively.

Let us consider below estimating the probabilistic parameter thresholds for 6 input variables $\theta _3$, $\theta _5$, $\theta _7$, $\theta _{10}$, $\theta _{11}$ and $\theta _{12}$, respectively. Before applying our proposed approach, we first show some reference results by estimating the conditional expectations $\mathbb {E}_{\varvec{\theta }_{-j}|\theta _j}[f_d(\varvec{\theta })]$ here. More precisely, for each considered input variable $\theta _j$, we estimate the conditional expectations $\mathbb {E}_{\varvec{\theta }_{-j}|\theta _j}[f_d(\varvec{\theta })]$ for all $d\in \{d_1,d_2,d_3\}$ by using the naive Monte Carlo average

$$\begin{aligned} \mathbb {E}_{\varvec{\theta }_{-j}|\theta _j}[f_d(\varvec{\theta })]\approx \frac{1}{N}\sum _{n=1}^{N}f_d(\varvec{\theta }_{-j}^{(n)},\theta _j) \end{aligned}$$

with large sample size $N=2^{18}$ for various values of $\theta _j$. Here, because of the multi-linearity of the functions $f_d$, the exact mean of an individual random variable can be substituted directly wherever available. The results are shown in Fig. 2. Except for the variable $\theta _{10}$, we can see that there exists exactly one intersection between every two different treatments: $(d_1,d_2)$, $(d_1,d_3)$ and $(d_2,d_3)$, where the intersection of the pair $(d_1,d_3)$ for $\theta _7$ exists beyond the range of this plot. It follows that the probabilistic parameter threshold $K_j$ consists of two elements for the variables except $\theta _{10}$. Regarding the variable $\theta _{10}$, the treatment $d_2$ always leads to a larger conditional expectation than the treatment $d_3$ and the corresponding probabilistic parameter threshold $K_{10}$ consists of only one element. We can use these results as a reference to see whether our proposed approach can search for the probabilistic parameter thresholds correctly.

### Experimental setup

We use our proposed Algorithm 2 with $M=1, T=N=10^4$ to estimate the threshold $K_j$, which means that we use only one sample of $\varvec{\theta }_{-j}$ at each iteration step in the second item of Algorithm 2. For the variables $\theta _{10},\theta _{11}$ and $\theta _{12}$, we consider the transformed variables $\log \theta _{10}, \mathrm {logit} (\theta _{11})$, and $\mathrm {logit} (\theta _{12})$ instead, respectively, for the iterations of stochastic approximation. In the first item of Algorithm 2, we generate $\theta _j^1$ from the marginal distribution of $\theta _j$. We set the sequence of step sizes to

$$\begin{aligned} \alpha _t = \frac{3\sigma (\theta _j)}{2\times 10^4\sqrt{t}}, \end{aligned}$$

where $\sigma (\theta _j)$ denotes the standard deviation for the marginal distribution of the variable $\theta _j$, and consider the averaged outputs $\Theta _j^t$ with $t=1,2,\ldots , T$ as a sequence of our threshold estimates. Regarding the variable $\theta _7$, we enlarge $\alpha _t$ by a constant factor so that the resulting estimate $\Theta _7^t$ converges within $T=10^4$ iteration steps. We carry out 20 independent runs for each considered variable.

### Convergence of pairwise estimates

**Figure 3:**
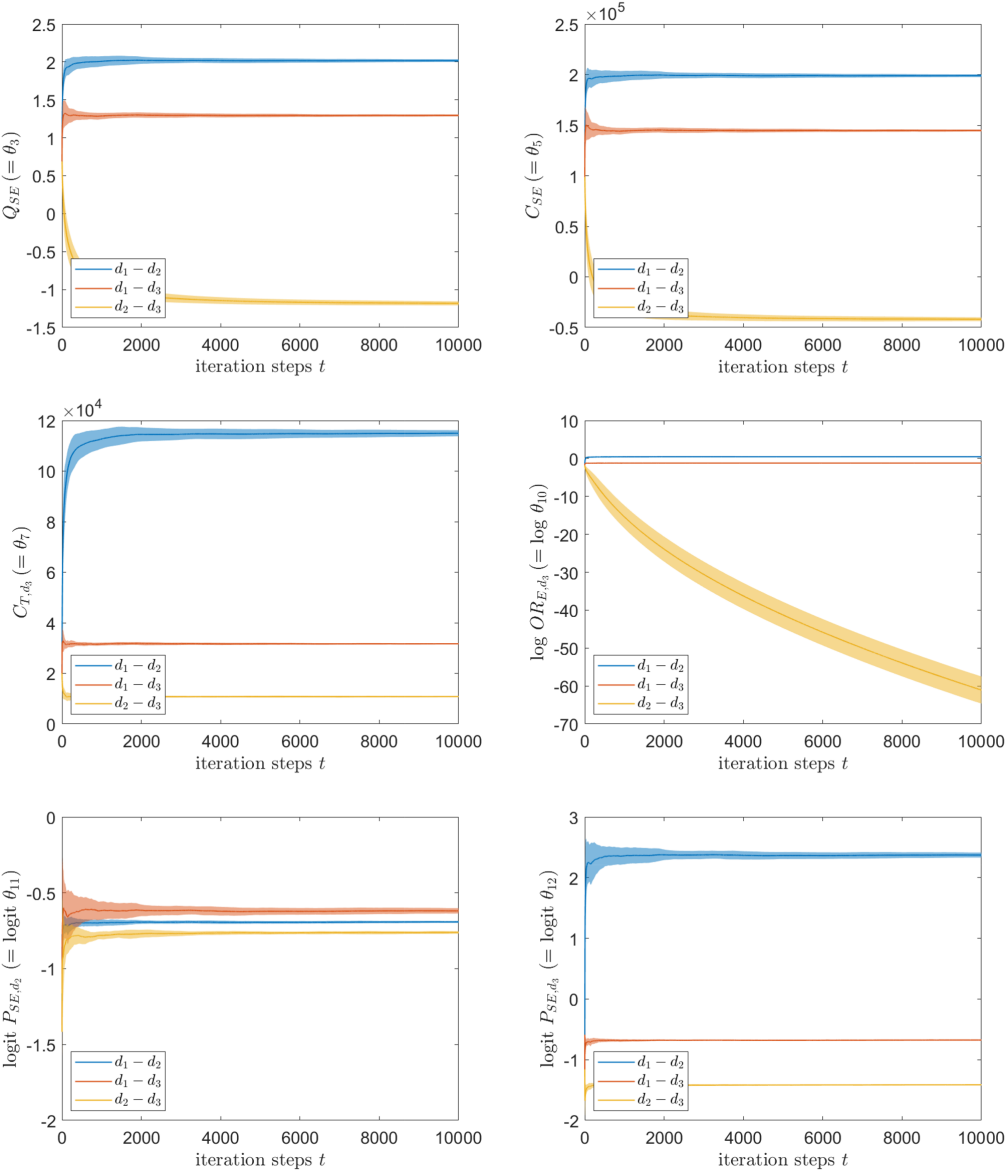
The pairwise probabilistic thresholds for the synthetic testcase found by the stochastic approximation with Polyak-Ruppert averaging as functions of iteration steps *t*. The results for $\theta _3$ (left top), $\theta _5$ (right top), $\theta _7$ (left middle), $\log \theta _{10}$ (right middle), $\mathrm {logit} (\theta _{11})$ (left bottom) and $\mathrm {logit} (\theta _{12})$ (right bottom) are shown respectively. For each pair of two treatments, the line and the shaded area represent the mean and its standard error estimated from 20 independent runs, respectively.

As the last paragraph shows how to set the input and the first item of Algorithm 2, here we discuss the results obtained from the second and third items of Algorithm 2. Figure 3 shows the convergence behaviors of the estimates $\Theta _j^t$ as functions of the iteration step *t*, obtained from the second item of Algorithm 2, for all the possible pairs $(d_1,d_2), (d_1,d_3)$ and $(d_2,d_3)$. Except for the pair $(d_2,d_3)$ for the variable $\log \theta _{10}$, the mean estimate from 20 independent runs converges to a value which agrees well with the intersection point shown in Fig. 2, and the standard error gets smaller with the convergence rate of approximately $t^{-1/2}$ as the iteration step *t* increases. These observations are exactly what we expect from the theory of stochastic approximation. Due to the convergence to a constant value, these estimates pass the third item of Algorithm 2 and can be subject to the last item.

Regarding the pair $(d_2,d_3)$ for the variable $\log \theta _{10}$, for which any intersection is not observed in Fig. 2, the mean estimate itself does not converge and the magnitude of the standard error stays almost the same along the iteration steps. This way, the resulting estimates do not pass the the third item of Algorithm 2 and we can infer that the set $K_{10}^{(d_2,d_3)}$ is empty. Interestingly, as clearly seen from the result for the pair $(d_1,d_2)$ for the variable $\theta _7$, the stochastic approximation method can find the pairwise threshold successfully even if it is located far from the initial estimate. It seems quite hard to get similar results if we only generate $\theta _7$ randomly from its marginal distribution as done in the existing approaches^27,28^.

### Estimated probabilistic thresholds

Finally we go to the fourth item of Algorithm 2. Table 2 summarizes the final estimates obtained from the stochastic approximation after $T=10^4$ iteration steps. Here the p-value is computed as follows. As an example, let us consider the estimated set for $K_j^{(d_1,d_2)}$. If it is not empty, we apply the one-sided *t*-test to the two pairs $(d_1,d_3)$ and $(d_2,d_3)$, which gives two p-values. By doing this for each of 20 independent runs, we have 20 of two p-values and finally we take the maximum value of the 20 p-values for each pair $(d_1,d_3)$ and $(d_2,d_3)$, respectively. In fact, the variables and the pairs in Table 2, which show the set of p-values as (1, 1), consistently yield (1, 1) for all 20 independent runs, which indicates that the corresponding estimate is contained in the threshold $K_j$. For some cases, the set of p-values is (0, 0), which clearly indicates that the corresponding estimate is *not* contained in $K_j$. The marginal case is found only for the pair $(d_1,d_2)$ for the variable $\log \theta _{10}$. As shown in Fig. 2, three intersections of the conditional expectations are close to each other, which makes it difficult to estimate $K_j$ correctly. Nevertheless, the p-values are small enough for the pair $(d_1,d_2)$, so that the estimated $K_{10}^{(d_1,d_2)}$ is properly discarded from the set $K_{10}$ with a sufficiently small significance level. Almost the same results are also obtained by applying one-sided Wilcoxon signed-rank test instead. This way our proposed algorithm can give estimates of the parameter probabilistic threshold which agree quite well with those expected from the reference results.

**Table 2:** The results of the pairwise probabilistic thresholds for the synthetic testcase found by the stochastic approximation with Polyak-Ruppert averaging after $10^4$ iterations.

Variable		$K_j^{(d_1,d_2)}$	$K_j^{(d_1,d_3)}$	$K_j^{(d_2,d_3)}$
$\theta _3$	Mean (std)	$\mathbf {2.02}$ (0.0176)	1.30 (0.0130)	$\mathbf {-1.18}$ (0.026)
	p-value	(1, 1)	(0, 0)	(1, 1)
$$\theta _5$$	Mean (std)	$$\mathbf {1.99\times 10^5}$$$$(1.31\times 10^3)$$	$$1.45\times 10^5$$$$(1.00\times 10^3)$$	$$\mathbf {-4.19\times 10^4}$$$$(1.88\times 10^3)$$
	p-value	(1, 1)	(0, 0)	(1, 1)
$$\theta _7$$	Mean (std)	$$\mathbf {1.15\times 10^5}$$$$(1.21\times 10^3)$$	$$3.17\times 10^4$$$$(2.29\times 10^2)$$	$$\mathbf {1.08\times 10^4}$$$$(2.49\times 10^2)$$
	p-value	(1, 1)	(0, 0)	(1, 1)
$$\log \theta _{10}$$	Mean (std)	$$\mathbf {0.45}$$$$(1.77\times 10^{-2})$$	$$-1.21$$$$(8.73\times 10^{-3})$$	Empty
	p-value	(1, 1)	(0, 0)	
$$\mathrm {logit} (\theta _{11})$$	Mean (std)	$$-0.69$$$$(6.27\times 10^{-3})$$	$$\mathbf {-0.62}$$$$(1.78\times 10^{-2})$$	$$\mathbf {-0.76}$$$$(8.09\times 10^{-3})$$
	p-value	$(5.36\times 10^{-4},9.38\times 10^{-8})$	(1, 1)	(1, 1)
$$\mathrm {logit} (\theta _{12})$$	Mean (std)	$$\mathbf {2.37}$$$$(4.16\times 10^{-2})$$	$$-0.66$$$$(5.57\times 10^{-3})$$	$$\mathbf {-1.41}$$$$(6.35\times 10^{-3})$$
	p-value	(1, 1)	(0, 0)	(1, 1)

The mean and its standard error are estimated from 20 independent runs, respectively. The p-values denote the maximum values for the one-sided *t*-test among 20 independent runs. The bold-typed numbers indicate that the corresponding elements are contained in the threshold $K_j$.

### Comparison with nested Monte Carlo

As a comparison, we estimate $K_j$ also by the nested Monte Carlo approach (Algorithm 1) with $M=10^4$ and $N=10^2$. Note that this choice of *M* and *N* is close to the one used by McCabe et al.^27^ and that the total cost for each variable is $MN=10^6$, which is already larger than that of our proposed approach. We have the following observations. Firstly, we cannot find any element in $K_j$ for the variables $\theta _3,\theta _5,\theta _7$ and $\log \theta _{10}$. This is expected from the fact that the outer samples are generated randomly from the marginal distribution for the nested Monte Carlo approach, whereas every threshold is located far from the region where the marginal probability distribution is concentrated in this case. Secondly, regarding the variable $\mathrm {logit} (\theta _{11})$, the resulting estimate is quite unstable in the sense that some runs (2 out of 20 runs) estimate that the threshold $K_{11}$ is empty, some (13 out of 20 runs) only estimate one of the two elements, and some (4 out of 20 runs) estimate the two elements but with larger variations than those by our proposed approach, and the remaining run mistakenly estimates that $K_{11}$ consists of three elements. The last mistaken estimation happens because the conditional expectations for at least two treatments are close to each other around the threshold and so the Monte Carlo estimation possibly returns a wrong treatment as the one which maximizes the conditional expectation. Finally, for the variable $\mathrm {logit} (\theta _{12})$, one of the two elements around $-1.41$, i.e., the intersection of the pair $(d_2,d_3)$, is estimated correctly for most runs (19 out of 20 runs), whereas the remaining run mistakenly estimates three distinct elements around $-1.41$. The mean and its standard error for the 19 runs are given by $-1.41$ and $1.13\times 10^{-2}$, respectively. Note that the mean agrees well with that obtained by our proposed approach, while the standard error is about twice larger for the nested Monte Carlo approach. No run can find the other element of $K_{12}$ around 2.37. This result clearly shows the superiority of our proposed approach.

### Decision switching probability

Using the results for the probabilistic thresholds, we can identify the intervals of $\theta _j$ where $d_{\mathrm {opt}}(\theta _j)$ is equal to $d_1,d_2$ and $d_3$, respectively, as shown in Table 3. As we have $d_{\mathrm {opt}}(\emptyset )=d_2$ for this model, the decision switching probability for a variable $\theta _j$ is given by

$$\begin{aligned} P_j = \mathbb {P}_{\theta _j}[d_{\mathrm {opt}}(\theta _j)=d_1]+\mathbb {P}_{\theta _j}[d_{\mathrm {opt}}(\theta _j)=d_3]. \end{aligned}$$

Each of two terms on the right-hand side can be computed from the marginal cumulative distribution function of $\theta _j$. The results are shown in the fifth column of Table 3. As a reference, we estimate the EVPPI for each variable by using the nested Monte Carlo estimator^17^ with $2^{18}$ outer samples for $\theta _j$ and $2^{10}$ inner conditional samples for $\varvec{\theta }_{-j}$, wherein the exact mean of a random variable is substituted directly whenever available. Note that the EVPI is estimated to be 4063.5 for this model^39^. Since every threshold for the variables $\theta _3,\theta _5,\theta _7$ and $\log \theta _{10}$ is located far from the region where the marginal probability distribution is concentrated, the corresponding decision switching probability is extremely small, and so is the EVPPI. On the contrary, one of the thresholds for both $\mathrm {logit} (\theta _{11})$ and $\mathrm {logit} (\theta _{12})$ is in the concentrated region, and so, knowing the exact value of $\mathrm {logit} (\theta _{11})$ or $\mathrm {logit} (\theta _{12})$ has some chance of changing the optimal treatment from $d_2$. In this case, the decision switching probability for $\mathrm {logit} (\theta _{12})$ is much larger than that for $\mathrm {logit} (\theta _{11})$, and so is the EVPPI, which indicates that the variable $\mathrm {logit} (\theta _{12})$ is more sensitive in choosing the optimal treatment.

**Table 3:** The results of the interval for the synthetic testcase over which the corresponding treatment $d\in \{d_1,d_2,d_3\}$ is optimal.

Variable	$d_{\mathrm {opt}}(\theta _j)=d_1$	$d_{\mathrm {opt}}(\theta _j)=d_2$	$d_{\mathrm {opt}}(\theta _j)=d_3$	DSP	EVPPI
$\theta _3$	$(2.02,\infty )$	$(-1.18, 2.02)$	$(-\infty ,-1.18)$	$5.57\times 10^{-40}$	0.0
$\theta _5$	$(1.99\times 10^5, \infty )$	$(-4.19\times 10^4, 1.99\times 10^5)$	$(-\infty ,-4.19\times 10^4)$	$2.17\times 10^{-23}$	0.0
$\theta _7$	$(1.15\times 10^5,\infty )$	$(1.08\times 10^4, 1.15\times 10^5)$	$(-\infty ,1.08\times 10^4)$	0.0	0.0
$\log \theta _{10}$	$(0.45,\infty )$	$(-\infty ,0.45)$	None	$1.36\times 10^{-19}$	0.00
$\mathrm {logit}(\theta _{11})$	$(-0.62,\infty )$	$(\infty ,-0.76)$	$(-0.76,-0.62)$	$2.17\times 10^{-2}$	54.3
$\mathrm {logit}(\theta _{12})$	$(2.37,\infty )$	$(-1.41,2.37)$	$(-\infty ,-1.41)$	$2.65\times 10^{-1}$	1308.9

The decision switching probability and the EVPPI are also shown in the fifth and sixth columns, respectively.

## Concluding remarks

In this paper we have developed an efficient pairwise stochastic approximation approach to estimate the probabilistic parameter threshold. Not only the standard theory on the convergence of stochastic approximation algorithms directly applies to our proposed approach but also the numerical experiments have confirmed that our proposed approach works quite well both for a simple synthetic testcase and a chemotherapy Markov model. Moreover, we have introduced a new measure called the decision switching probability for probabilistic sensitivity analysis in the context of health economic evaluations, or more broadly, decision-making under uncertainty, which can deliver a complementary information to the existing decision-theoretic probabilistic sensitivity measure EVPPI.

The following issues are left for future research:

- As with the existing methods^27,28^, our proposed approach applies only to the probabilistic parameter threshold for a single input variable $\theta _j$. An extension to the multivariate case is interesting but does not seem straightforward.
- Although we have not discussed in this paper, it is clear that the decision switching probability can be also defined for the sample information and used as a complementary measure to the *expected information of sample information*^39,41–44^. We need further investigation on how to efficiently estimate the decision switching probability for sample information, as our present approach using the probabilistic parameter threshold and the marginal probability distribution is not straightforward to extend.

## Supplementary Information

Supplementary Information.
