# Supplementary material for: Probabilistic threshold analysis by pairwise stochastic approximation for decision-making under uncertainty
Source: Sci Rep. 2021 Oct 4;11:19671. doi: 10.1038/s41598-021-99089-z (PMC8490445; doi:10.1038/s41598-021-99089-z)
Supplement: Supplementary file 1 — Supplementary Information. [file 41598_2021_99089_MOESM1_ESM.pdf]

Supplementary Information: Probabilistic threshold analysis by pairwise stochastic approximation for decision-making under uncertainty

Takashi Goda and Yuki Yamada

May 26, 2021

1 EVPPI versus decision switching probability

Here we show an example of the two-alternative decision models for which the larger EVPPI does not necessarily mean the larger decision switching probability. Let $D = \{d_1, d_2\}$ and $\boldsymbol{\theta} = (\theta_1, \theta_2)$ with θ_1 and θ_2 independently following the standard normal distribution. The functions f_{d_1} and f_{d_2} are given by

$$\begin{aligned} f_{d_1}(\boldsymbol{\theta}) &= 0, \\ f_{d_2}(\boldsymbol{\theta}) &= \theta_1^2 - \theta_2^2 - \alpha, \end{aligned}$$

respectively, with some constant $0 < \alpha < 1$. Since

$$\mathbb{E}_{\boldsymbol{\theta}}[f_{d_1}(\boldsymbol{\theta})] = 0 > \mathbb{E}_{\boldsymbol{\theta}}[f_{d_2}(\boldsymbol{\theta})] = 1 - 1 - \alpha = -\alpha,$$

we have

$$d_{\mathrm{opt}}(\emptyset) = d_1 \quad \text{and} \quad \max_{d \in D} \mathbb{E}_{\boldsymbol{\theta}}\left[f_d(\boldsymbol{\theta})\right] = \mathbb{E}_{\boldsymbol{\theta}}[f_{d_1}(\boldsymbol{\theta})] = 0.$$

Let us consider the partial perfect information on θ_1. As we have

$$\mathbb{E}_{\theta_2|\theta_1}[f_{d_1}(\boldsymbol{\theta})] = 0 \quad \text{and} \quad \mathbb{E}_{\theta_2|\theta_1}[f_{d_2}(\boldsymbol{\theta})] = \theta_1^2 - 1 - \alpha,$$

the conditionally optimal option becomes

$$d_{\mathrm{opt}}(\theta_1) = \begin{cases} d_1 & \text{for } -\sqrt{1+\alpha} < \theta_1 < \sqrt{1+\alpha}, \\ d_2 & \text{for } \theta_1 < -\sqrt{1+\alpha} \text{ or } \theta_1 > \sqrt{1+\alpha}, \end{cases}$$

where $d_{\mathrm{opt}}(\theta_1)$ is not unique for $\theta_1 = \pm\sqrt{1+\alpha}$. Now the decision switching probability for θ_1 is given by

$$\begin{aligned} P_1 &= \mathbb{P}_{\theta_1}\left[d_{\mathrm{opt}}(\theta_1) \neq d_{\mathrm{opt}}(\emptyset)\right] = \mathbb{P}_{\theta_1}\left[d_{\mathrm{opt}}(\theta_1) = d_2\right] \\ &= \mathbb{P}_{\theta_1}\left[\theta_1 < -\sqrt{1+\alpha}\right] + \mathbb{P}_{\theta_1}\left[\theta_1 > \sqrt{1+\alpha}\right] \\ &= 1 - \mathrm{erf}\left(\sqrt{\frac{1+\alpha}{2}}\right), \end{aligned}$$

where erf denotes the error function, while the EVPPI for θ_1 is given by

$$\begin{aligned} \mathrm{EVPPI}_{\theta_1} &= \mathbb{E}_{\theta_1}\left[\max\left(0, \theta_1^2 - 1 - \alpha\right)\right] - 0 \\ &= e^{-(1+\alpha)/2}\sqrt{\frac{2(1+\alpha)}{\pi}} - \alpha\left(1 - \mathrm{erf}\left(\sqrt{\frac{1+\alpha}{2}}\right)\right). \end{aligned}$$

A similar computation leads to the decision switching probability for θ_2:

$$P_2 = \mathrm{erf}\left(\sqrt{\frac{1-\alpha}{2}}\right),$$

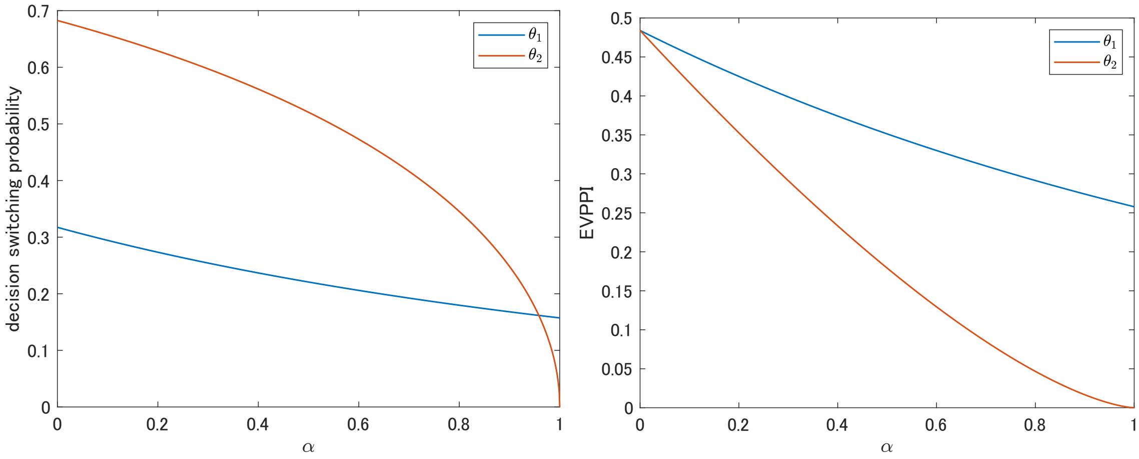

Supplementary Figure 1: The decision switching probability (left) and the EVPPI (right) for θ_1 and θ_2 as functions of α.

and the EVPPI for θ_2:

$$\mathrm{EVPPI}_{\theta_2} = e^{-(1-\alpha)/2}\sqrt{\frac{2(1-\alpha)}{\pi}} - \alpha\,\mathrm{erf}\left(\sqrt{\frac{1-\alpha}{2}}\right).$$

There results are compared in Supplementary Figure 1. The left panel shows the behaviors of the decision switching probability for θ_1 (in blue) and θ_2 (in orange) as functions of $\alpha \in (0,1)$, respectively, whereas the right panel shows those of the EVPPI. The decision switching probability monotonically decreases both for θ_1 and θ_2 as α increases. It is important that the decision switching probability for θ_2 is much larger than that for θ_1 when α is small. This means that knowing the value of θ_2 exactly has a higher chance of changing the optimal option from d_1 to d_2. On the contrary, knowing the exact value of θ_1 has a high chance of keeping the optimal option unchanged, making the partial prefect information useless in terms of decision making. The two curves intersect at $\alpha \approx 0.958362$, beyond which the decision switching probability for θ_1 becomes larger.

The EVPPI decreases both for θ_1 and θ_2 as α increases. Such a behavior is consistent with the decision switching probability. However, for all $\alpha \in (0,1)$, the EVPPI for θ_1 is larger than that for θ_2, which is opposite to the decision switching probability when $\alpha < 0.958362$. From these results, we see that knowing the exact value of θ_1 has a low chance of changing the optimal option from d_1 to d_2 but an increment of the expected cost-effectiveness gained from such decision switching is more significant than knowing the exact value of θ_2. Through this example, we can conclude that the larger EVPPI does not necessarily mean the larger decision switching probability and vice versa.

2 Additional numerical experiments

2.1 Problem setting

Here we test our proposed approach for an additional example introduced by Heath and Baio [2]. The model compares two different chemotherapy treatments: a standard of care (d_1) and a novel treatment (d_2). The cost and the quality-adjusted life year (QALY) for each treatment are modelled by a homogeneous, discrete-time Markov process with four states (recorvery, home care, hospital care, and death) depending on whether patients experience side effects or not. The difference between two treatments is in the probability of experiencing side effects. Here π_{d_1} denotes the probability of experiencing side effects for the standard of care, and ρ denotes the relative percentage in the number of side effects for the new treatment from that for the standard of care. That is, the probability of experiencing side effects for the new treatment is given by the product $\rho\pi_{d_1}$. The input vector $\boldsymbol{\theta}$ consists of 12 random variables π_{d_1}, ρ, Γ_1, Γ_2, λ_1, λ_2, C_{Death}, C_{Hosp}, C_{Home}, Q_{Recov}, Q_{Hosp}, Q_{Home}, which are denoted by $\theta_1, \ldots, \theta_{12}$, respectively, in this order. The model contains three constants $C_{d_1}, C_{d_2}, \lambda$, while two parameters γ_1 and

Description	Parameter	Distribution
Probability of side effects on treatment $d = d_1$	$\pi_{d_1}\,(\theta_1)$	$\mathrm{Beta}(28, 85)$
Reduction in side effects on treatment $d = d_2$	$\rho\,(\theta_2)$	$N\,(0.65, 0.01)$
Probability of hospitalization for 15 days	$\Gamma_1\,(\theta_3)$	$\mathrm{Beta}(11, 18)$
Probability of dying for 15 days	$\Gamma_2\,(\theta_4)$	$\mathrm{Beta}(2, 13)$
Probability of hospitalization per day	$\gamma_1 = \Gamma_1/15$	Derived from Γ_1
Probability of dying per day	$\gamma_2 = \Gamma_2/15$	Derived from Γ_2
Probability of recovery from home care	$\lambda_1\,(\theta_5)$	$\mathrm{Beta}(5.12, 6.26)$
Probability of recovery from hospital care	$\lambda_2\,(\theta_6)$	$\mathrm{Beta}(3.63, 6.74)$
Cost of treatment $d = d_1$	C_{d_1}	110 (constant)
Cost of treatment $d = d_2$	C_{d_2}	420 (constant)
Cost of death	$C_{\mathrm{Death}}\,(\theta_7)$	$\text{log-normal}(8.33, (0.13)^2)$
Cost of hospital care	$C_{\mathrm{Hosp}}\,(\theta_8)$	$\text{log-normal}(8.77, (0.15)^2)$
Cost of home care	$C_{\mathrm{Home}}\,(\theta_9)$	$\text{log-normal}(7.74, (0.039)^2)$
QALY after recovery	$Q_{\mathrm{Recov}}\,(\theta_{10})$	$\mathrm{Beta}(18.23, 0.372)$
QALY for hospital care	$Q_{\mathrm{Hosp}}\,(\theta_{11})$	$\mathrm{Beta}(0.87, 3.47)$
QALY for home care	$Q_{\mathrm{Home}}\,(\theta_{12})$	$\mathrm{Beta}(5.75, 5.75)$
Willingness-to-pay	λ	30,000 (constant)

Supplementary Table 1: List of the input parameters for the Markov chemotherapy model.

γ_2 are defined as functions of Γ_1 and Γ_2, respectively. A detailed description on these model inputs is provided in Supplementary Table 1. For each treatment $d \in D$, the cost-effectiveness is defined by

$$f_d(\boldsymbol{\theta}) = -C_d + \frac{1}{15 \times 10^3} \sum_{i=0}^{15} \begin{pmatrix} SE_d \\ 0 \\ 0 \\ 0 \end{pmatrix} \boldsymbol{M}^i \begin{pmatrix} \lambda Q_{\mathrm{Home}} - C_{\mathrm{Home}} \\ \lambda Q_{\mathrm{Hosp}} - C_{\mathrm{Hosp}} \\ \lambda Q_{\mathrm{Recov}} \\ -C_{\mathrm{Death}} \end{pmatrix},$$

with $SE_{d_1} \sim \mathrm{Bin}(10^3, \pi_{d_1})$ and $SE_{d_2} \sim \mathrm{Bin}(10^3, \rho\pi_{d_1})$ being the conditionally independent random variables given π_{d_1}, where $\mathrm{Bin}(n, p)$ denotes the Binomial distribution with n independent trials and success probability p for each trial. Moreover, the matrix $\boldsymbol{M} \in \mathbb{R}_{\geq 0}^{4\times 4}$ denotes the transition matrix of the time-homogeneous Markov process given by

$$\boldsymbol{M} = \begin{pmatrix} (1-\lambda_1)(1-\gamma_1) & \gamma_1 & \lambda_1(1-\gamma_1) & 0 \\ 0 & (1-\lambda_2)(1-\gamma_2) & \lambda_2(1-\gamma_2) & \gamma_2 \\ 0 & 0 & 1 & 0 \\ 0 & 0 & 0 & 1 \end{pmatrix}.$$

It is clear that the functions f_{d_1} and f_{d_2} are non-linear with respect to some of input variables, π_{d_1}, ρ, Γ_1, Γ_2, λ_1 and λ_2, whereas they remain linear with respect to the other variables. Even through the input variables are assumed mutually independent, such non-linearity of the functions makes it quite hard to compute the probabilistic parameter threshold exactly. Nevertheless, since only two treatments are compared in this model, the probabilistic parameter threshold can be estimated by solving a single root-finding problem without any need for hypothesis testing, that is, the fourth item in Algorithm 2 (from the main text [1]) can be skipped.

2.2 Results and discussion

We consider estimating the probabilistic parameter thresholds for 4 input variables θ_1, θ_2, θ_5 and θ_6, respectively. We use Algorithm 2 (from the main text [1]) without the fourth step and with $M = 1, T = 10^4$ to estimate the threshold K_j. In the first item of Algorithm 2, we generate θ_j^1 from the marginal

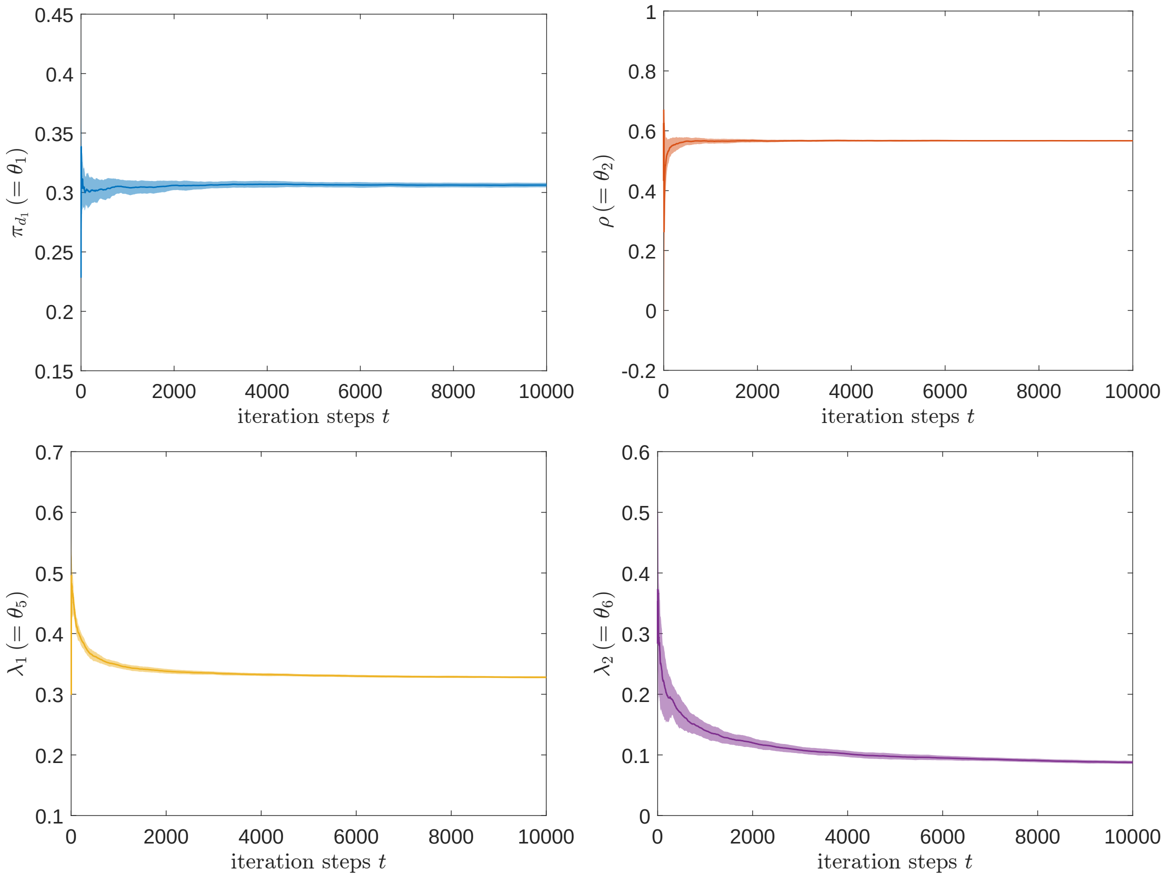

Supplementary Figure 2: The convergence behaviors of the pairwise probabilistic threshold estimates for the Markov chemotherapy model. The results for θ_1 (left top), θ_2 (right top), θ_5 (left bottom) and θ_5 (right bottom) are shown respectively.

distribution of θ_j. We set the sequence of step sizes to

$$\alpha_t = \frac{3\sigma(\theta_j)}{50\sqrt{t}},$$

where $\sigma(\theta_j)$ denotes the standard deviation for the marginal distribution of the variable θ_j. As the variables θ_1, θ_5 and θ_6 are defined over the interval $[0, 1]$, if the recursion (2) in the main text [1] yields $\theta_j^{t+1} < 0$ or $\theta_j^{t+1} > 1$, we put $\theta_j^{t+1} = 0$ or $\theta_j^{t+1} = 1$, respectively. Moreover, as the product $\theta_1\theta_2\,(= \rho\pi_{d_1})$ is used as a parameter to generate a binomial random variable SE_{d_2}, we must have $0 \leq \theta_1\theta_2 \leq 1$. In our experiments, instead of restricting the support of θ_2, we simply consider $SE_{d_2} \equiv 0$ if $\theta_1\theta_2 < 0$ and $SE_{d_2} \equiv 10^3$ if $\theta_1\theta_2 > 0$. The averaged outputs Θ_j^t are considered as a sequence of our threshold estimates. We carry out 20 independent runs for each considered variable.

Supplementary Figure 2 shows the convergence behaviors of the estimates Θ_j^t as functions of t, obtained from the second item of Algorithm 2. For all 20 runs, the estimates converge to a similar value, indicating that the threshold K_j consists of only one element. The standard error decays with the convergence rate of approximately $t^{-1/2}$, or even better for the variables θ_2 and θ_6. This way our proposed approach works quite well not only for the simple synthetic testcase but also for such a complicated Markov model.

The second column of Supplementary Table 2 summarizes the final estimates for the threshold K_j obtained by our proposed approach after $T = 10^4$ iteration steps. Using these results, the intervals of θ_j where $d_{\text{opt}}(\theta_j)$ is equal to d_1 and d_2 are identified as shown in the third and fourth columns, respectively. For this model we have $d_{\text{opt}}(\emptyset) = d_1$, so that the decision switching probability for a variable θ_j is given by

$$P_j = \mathbb{P}_{\theta_j}[d_{\text{opt}}(\theta_j) = d_2],$$

variable	K_j	$d_{\mathrm{opt}}(\theta_j) = d_1$	$d_{\mathrm{opt}}(\theta_j) = d_2$	DSP	EVPPI
θ_1	0.306 (1.86×10^{-3})	$[0, 0.306)$	$(0.306, 1]$	7.95×10^{-2}	1.68
θ_2	0.567 (1.86×10^{-3})	$(0.567, \infty)$	$(-\infty, 0.567)$	2.02×10^{-1}	8.17
θ_5	0.328 (1.68×10^{-3})	$(0.328, 1]$	$[0, 0.328)$	2.07×10^{-1}	16.73
θ_6	0.087 (2.60×10^{-3})	$(0.087, 1]$	$[0, 0.087)$	1.32×10^{-2}	0.03

Supplementary Table 2: A summary on the results for the Markov chemotherapy model.

which can be computed by the marginal cumulative distribution function. The resulting values are shown in the fifth column. The variable θ_6 yields the minimum decision switching probability, whereas the variables θ_2 and θ_5 yield the approximately equal probabilities about 0.2. As a reference, we estimate the EVPPI for each variable by using the nested Monte Carlo estimator with 2^{18} outer samples for θ_j and 2^{10} inner samples for $\boldsymbol{\theta}_{-j}$. Note that the EVPI is estimated to be 36.81 by using the standard Monte Carlo estimator with 2^{24} random samples for $\boldsymbol{\theta}$. The estimated EVPPI values are shown in the sixth column. The variables θ_1 and θ_6 for which the decision switching probabilities are small yield the relatively small EVPPI values. Although the decision switching probabilities for θ_2 and θ_5 are almost the same, the EVPPI for θ_5 is twice larger than that for θ_2. It is interesting to see that a variable with larger EVPPI does not necessarily have a larger decision switching probability not only for an artificial model considered in Supplementary Information 1 but also for a more realistic model for health economic evaluations.

References

[1] T. Goda and Y. Yamada. Probabilistic threshold analysis by pairwise stochastic approximation for decision-making under uncertainty, 2021.

[2] Anna Heath and Gianluca Baio. Calculating the expected value of sample information using efficient nested Monte Carlo: A tutorial. *Value in Health*, 21(11):1299–1304, 2018.
